# Quantification of porcine myocardial perfusion with modified dual bolus MRI – a prospective study with a PET reference

**DOI:** 10.1186/s12880-019-0359-8

**Published:** 2019-07-26

**Authors:** Minna Husso, Mikko J. Nissi, Antti Kuivanen, Paavo Halonen, Miikka Tarkia, Jarmo Teuho, Virva Saunavaara, Pauli Vainio, Petri Sipola, Hannu Manninen, Seppo Ylä-Herttuala, Juhani Knuuti, Juha Töyräs

**Affiliations:** 1 Diagnostic Imaging Center, Kuopio University Hospital, PO Box 100, 70029 Kuopio, KYS Finland; 2 Department of Applied Physics, University of Eastern Finland, Kuopio, Finland; 3 A.I. Virtanen Institute for Molecule Sciences, University of Eastern Finland, Kuopio, Finland; 4 Turku PET Centre, Turku University Hospital and University of Turku, Turku, Finland; 5 Department of Medical Physics, Turku University Hospital, Turku, Finland; 6 Heart Center and Gene Therapy Unit, Kuopio University Hospital, Kuopio, Finland; 7 School of Information Technology and Electrical Engineering, The University of Queensland, Brisbane, Australia

**Keywords:** Magnetic resonance imaging (MRI), Myocardial perfusion imaging, Modified dual bolus method, Quantification, Arterial input function, Positron emission tomography (PET)

## Abstract

**Background:**

The reliable quantification of myocardial blood flow (*MBF*) with MRI, necessitates the correction of errors in arterial input function (AIF) caused by the T1 saturation effect. The aim of this study was to compare *MBF* determined by a traditional dual bolus method against a modified dual bolus approach and to evaluate both methods against PET in a porcine model of myocardial ischemia.

**Methods:**

Local myocardial ischemia was induced in five pigs, which were subsequently examined with contrast enhanced MRI (gadoteric acid) and PET (O-15 water). In the determination of *MBF*, the initial high concentration AIF was corrected using the ratio of low and high contrast AIF areas, normalized according to the corresponding heart rates. *MBF* was determined from the MRI, during stress and at rest, using the dual bolus and the modified dual bolus methods in 24 segments of the myocardium (total of 240 segments, five pigs in stress and rest). Due to image artifacts and technical problems 53% of the segments had to be rejected from further analyses. These two estimates were later compared against respective rest and stress PET-based *MBF* measurements.

**Results:**

Values of *MBF* were determined for 112/240 regions. Correlations for *MBF* between the modified dual bolus method and PET was *r*_*s*_ = 0.84, and between the traditional dual bolus method and PET *r*_*s*_ = 0.79. The intraclass correlation was very good (ICC = 0.85) between the modified dual bolus method and PET, but poor between the traditional dual bolus method and PET (ICC = 0.07).

**Conclusions:**

The modified dual bolus method showed a better agreement with PET than the traditional dual bolus method. The modified dual bolus method was found to be more reliable than the traditional dual bolus method, especially when there was variation in the heart rate. However, the difference between the *MBF* values estimated with either of the two MRI-based dual-bolus methods and those estimated with the gold-standard PET method were statistically significant.

**Electronic supplementary material:**

The online version of this article (10.1186/s12880-019-0359-8) contains supplementary material, which is available to authorized users.

## Background

Accurate quantification of myocardial perfusion is important in the diagnosis of cardiac diseases. There are several contrast enhanced MRI methods available for measuring myocardial perfusion [1]. The determination of myocardial blood flow (*MBF*) with contrast enhanced MRI necessitates monitoring the concentration of contrast agent in blood and myocardium. The range of contrast agent concentrations in blood is much higher than that in tissue. Quantitative myocardial perfusion imaging is based on the assumption that the MRI signal increases linearly with the contrast agent concentration but this linear relationship is not valid at high contrast agent concentrations. Therefore, one major challenge in the application of contrast agent enhanced MRI is to control the T1 effect [2]. This effect causes non-linearity of the signal intensity (SI) in MRI images, and underestimation of the amplitude of the arterial input function (AIF). This is important since underestimation of AIF leads to incorrect determination of *MBF*. One solution to this problem has been to convert the signal intensity to contrast agent concentration using the known nonlinear relationship between SI and contrast agent concentration. Although this method is widely used with human patients [3, 4], it is also known to be heavily dependent on haematocrit [5]. An alternative solution is to use low contrast agent concentration, in order to avoid the non-linearity of the input function, as Jerosch-Herold et al., Christian et al. and Wilke et al. in their studies with pigs [6–8]. Unfortunately, this may lead to low signal-to-noise ratios in areas with low perfusion [9]. Gatehouse et al. introduced a dual-sequence method with human patients [10] in which two images are acquired during one heartbeat. First, a low-resolution AIF image with low T1 sensitivity and an approximately linear response over a large concentration range is acquired. The other image is acquired to determine signal intensity in myocardium. This elegant method was tested by Sánchez-González et al. using phantom measurements and pigs [11]. The dual bolus method, introduced by Köstler et al. with human patients [12] and validated by Christian et al. with dogs [13] is based on a different approach to resolve the non-linearity of AIF. The method is based on two separate contrast agent injections during the dynamic imaging sequence. First injection (pre-bolus) contains diluted contrast agent. The low concentration of contrast agent is used to ensure the linear correlation between the concentration of contrast agent and signal intensity in MR image and therefore the accurate determination of AIF. After that, another injection with high concentration contrast agent bolus is performed for the determination of tissue residue curve. Unfortunately, this method cannot take into account variation in kinetics of the contrast agent injections, which may be due to differences in the circulation (e.g. heart rate variations) and/or to a different dispersion of contrast agent bolus due to variable intrathoracic pressure during inspiration. To overcome this problem, a modified dual bolus method [14] was introduced with human patients. The modified dual bolus method uses the low concentration pre-bolus to correct the AIF of the high concentration contrast agent injection. Therefore, both the corrected AIF and the residue curve of the same primary high concentration injection can be used to determine the *MBF*, avoiding issues arising from separately measured AIF. However, this technique has not been validated by comparing it with the current gold standard i.e. positron emission tomography (PET) perfusion imaging.

PET is the gold standard in the determination of *MBF* [15]. The use of radioactive ^15^O-water makes it possible to quantify absolute perfusion. However, the challenge in the use of PET is its availability, because the production of ^15^O-water requires an on-site cyclotron. Furthermore, in PET, exposure to ionizing radiation cannot be avoided.

The aim of this study was to compare *MBF* determined by the traditional dual bolus and the modified dual bolus methods and evaluate them both against PET in a porcine model of cardiac ischemia.

## Methods

### The animal model

Five female domestic pigs (age 3–4 months), weight range 28–39 kg, were investigated. All animal experiments were approved by the National Animal Experiment Board (licence no. ESAVI-2012-001932), and conform to the Directive 2010/63/EU of the European Parliament. All animals were examined with MRI and PET. Local myocardial ischemia was induced in the pig heart using a constricted bare metal stent, which was positioned into the left anterior descending artery [16]. Complete occlusion of the bare metal stent was prevented by anticoagulation therapy: dual-antiplatelet regimen (DAPT) during the experiment, ASA (100 mg/day po), clopidogrel (75 mg/day po), and enoxaparin (30 mg/day sc) were continued for the entire duration of the experiment. The pigs were imaged within 12–24 days after the operation. Animals were anaesthetized with intramuscular administration of midazolam 1 mg/kg (Midazolam Hameln, Hameln, Germany) and xylazine 4 mg/kg (Rompun vet, Leverkusen, Germany), connected to a respirator and ventilated mechanically (tidal volume 8–10 ml/kg, frequency 14–18 1/min, Dräger Oxylog 3000, Lübeck, Germany). An ear vein was cannulated with a 22G venous catheter and anesthesia was maintained with an intravenous infusion of propofol (10–50 mg/kg/h, B. Braun Melsungen AG, Melsungen, Germany) combined with fentanyl (4–8 μg/kg/h, Hameln Pharmaceuticals GmbH, Hameln, Germany). The anaesthesia was kept stable during the whole imaging procedure.

### PET imaging protocol

A Philips Ingenuity TF PET/MR (Philips, Amsterdam, Netherlands) scanner was used for both PET and MR imaging during the same session maintaining the same positioning between the modalities. The scanner is composed of a PET gantry and a 3 T MRI gantry, which are assembled opposite to each other and connected together via a single patient table [17]. This scanner maintains the same positioning during PET and MR imaging, but does not acquire images simultaneously. Animals first underwent a myocardial perfusion PET study with ^15^O-water under pharmacologic stress and subsequently at rest, after their hemodynamics had returned to the baseline level. The ^15^O-water (Radiowater Generator, Hidex Oy, Turku, Finland) was injected intravenously via the ear vein as a 15 s bolus. The injected radioactivity was 790 ± 74 MBq (range 628–879 MBq). The dynamic scanning started at the same time as the injection. The acquisition frames were as follows: 26 × 5 s, 3 × 10 s, 3 × 20 s, 4 × 30 s (total duration 5 min 40 s). The duration of the early frames was set to produce AIF with a good temporal resolution and the late frames with sufficient signal statistics taking decay of ^15^O into account. Pharmacologic stress was induced with an intravenous injection of adenosine at the rate of 500 μg/kg/min (Adenosin Life Medical, Life Medical Sweden AB, Stocksund, Sweden) combined with phenylephrine (5 μg/kg/min, Fenylefrin Abcur, Abcur AB, Helsingborg, Sweden), starting 2 min prior to PET imaging and continuing throughout the stress study to induce myocardial hyperemia. The adenosine dose of 500 μg/kg/min was chosen to achieve maximal vasodilatation [18]. To maintain blood pressure, the a1-adrenoceptor agonist phenylephrine was co-infused (5 mg/kg/min i.v., Pharmacy Erasmus MC, Rotterdam, The Netherlands) with adenosine.

### MR imaging protocol

**Fig. 1:**
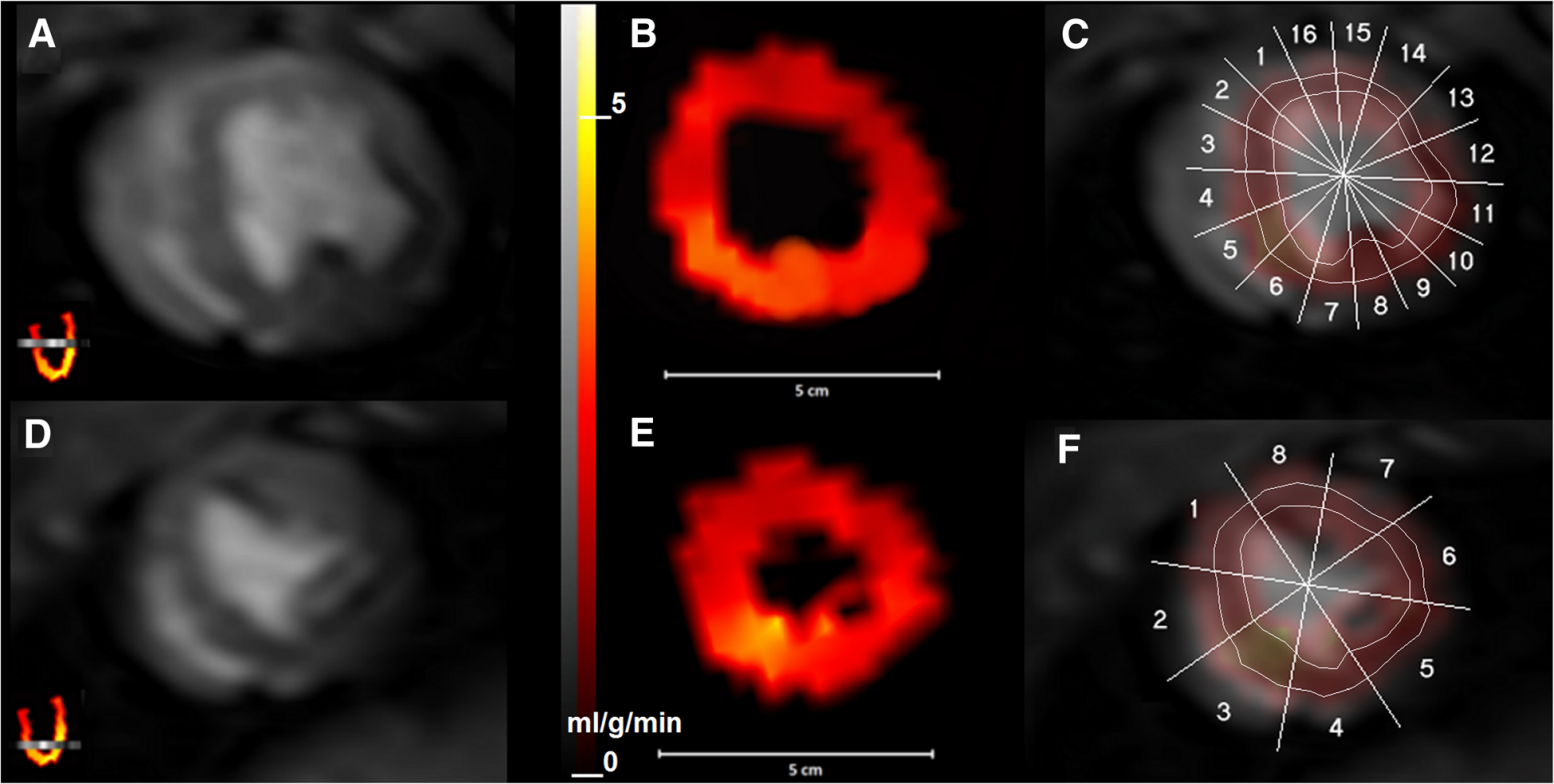
MRI-, PET- and co-registered images of the short axis slice. The mid-ventricular short axis slice and (upper row) and the apical short axis slice (lower row). **a** and **d** MR images, **b** and **e** PET images, **c** and **f** co-registered short axis MRI- and PET-images with ROIs. Orientation of the MRI slices is indicated in long axis PET images in the lower left corners of the MR images. MR images were acquired using the T1-TFE sequence. Contrast agent can be seen in the right and left ventricles

After PET imaging, the pig was removed from the PET gantry, a cardiac array surface coil was positioned over the heart region and MR imaging was carried out. A four-lead vectocardiogram was used for cardiac gating. Contrast-enhanced myocardial perfusion imaging was performed using the imaging parameters described in Table 1. Two parallel 8 mm short axis slices with an 8 mm gap were imaged. The slices in the dynamic series were adjusted to avoid the metal stent. The apical slice was set 16 mm from the apex, and the mid-ventricular slice 8 mm from the apical slice (Fig. 1 a and d). The scan repetition time was automatically adjusted according to cardiac gating to be the shortest possible value depending on the heart rate during the scan (typically between 0.58 s - 1.38 s). These parameters enabled the imaging of both slices during each heartbeat.

**Table 1:** MR imaging parameters

Sequence	2D saturation recovery segmented gradient recalled echo (T1-TFE)
TR^a^	‘shortest’ (typically 3.3 ms)
TE^b^	‘shortest’ (typically 1.6 ms)
Saturation recovery time	150 ms
FOV^c^	350 mm × 350 mm
Acquisition matrix	92 × 128
Flip angle^d^	20°

^a^TR Time to repetition

^b^TE Time to echo

^c^FOV Field of view

^d^The flip angle was chosen to achieve maximal T1-weighting

The first, low concentration pre-bolus contrast agent was injected after the 3rd frame of the dynamic image series was acquired. The low concentration dilution was prepared by adding 5 ml of gadoteric acid (Dotarem, 0.5 mmol/ml Guerbet LLC, Bloomington, IN, USA) into 100 ml of 0.9% saline (concentration ratio: 5 ml/105 ml = 1/21) and then 0.1 ml/kg of this dilution was injected as an intravenous bolus into the ear vein manually as quickly as possible, thereafter 15 ml of saline was injected manually for flushing. Dynamic MR acquisition was repeated continuously for every cardiac cycle during 60 heart beats. The actual perfusion series was carried out immediately after the pre-bolus imaging in a similar fashion with the exception that contrast agent was administered without dilution. Contrast agent (0.1 ml/kg = 0.05 mmol/kg) was chosen to achieve a strong enhancement in myocardium while avoiding significant saturation effects [19]. The dynamic first-pass imaging procedure described above was carried out in both the stressed and rest conditions similarly to the PET perfusion imaging procedure. The pigs were ventilated normally during the PET and MR perfusion imaging, i.e. imaging was performed during free-breathing. Immediately after the imaging studies, the animals were sacrificed by intravenous injection of potassium chloride (B. Braun Medical Oy, Helsinki, Finland). During the injection of potassium chloride the animals were under the same anaesthesia than during the imaging procedure.

### Image analysis

PET image analysis was carried out with Carimas software (Version 2.9, PET centre, Turku Finland 2014) [20]. PET data was volumetrically sampled, and a region-of-interest (ROI) covering the whole left ventricle was applied to the dynamic imaging series in order to obtain myocardial time-activity curves (TAC). Arterial input function was obtained for a cylindrical volume of interest centered in the basal portion of the LV. The segmental average LV *MBF* was determined based on ^15^O-water images using the conventional single-compartment model [21].

MR Images were analysed with the Carimas software. At first, PET and MR images were semi-automatically co-registered. Co-registration was performed using the orientation parameters (x-y-z) of MRI- and PET images. Minor manual corrections were made when necessary. In the determination of the myocardial perfusion, ROIs at the myocardium and left ventricle were drawn onto the MR images. The mid-ventricular slice was divided into 16, and the apical slice into eight similarly sized regions. Then, ROIs were drawn inside the segment boundaries in the mid-myocardial region. The cavity of the left and right ventricles were carefully avoided (Fig. 1c and f).

Because the PET data was in 3-D, the ROIs in MR images were converted into volumes of interest (VOI). The dynamic MR image series were checked frame by frame to ensure that the ROIs were accurately drawn on the myocardium. In case the ROIs were out of myocardium due to respiratory motion, the ROI location was manually corrected. Subsequently, signal intensity (SI) – time curves for each VOI were obtained. Next, the VOIs were copied into the co-registered PET images. The voxel size in PET images was 4 mm × 4 mm × 4 mm, which was different from that of MR images (3.8 mm × 2.7 mm × 8 mm). For that reason, trilinear interpolation was used when 2-D ROIs were converted into volumes of interest and copied onto the PET images. Values of *MBF* for each VOI were determined from PET images (Fig. 1 b and e).

SI – time curves of MR images were processed in Matlab (v. 2014b, The MathWorks, Natick, MA, US). The stress MRI study of one pig (#3) had to be rejected (24 curves), because it failed technically. Therefore, altogether 216 residue curves were initially obtained. Furthermore, dark rim artifact [22] was unfortunately common. This phenomenon was seen as the SI in the myocardium was decreased simultaneously with the arrival of the contrast agent in the left ventricle. First, the mean baseline values of signal intensity of tissue SI-curves during the time 0–7 s of image series were calculated. If the SI of tissue enhancement curve dropped below the baseline at the arrival of the contrast agent (and subsequent increase of SI), the curve was rejected from further analyses (104 curves). These rejected curves were mainly from the septum area (shown detailed in Additional file 1: Table S1.1). The baseline was determined as a mean of myocardial SI before the contrast agent arrival into the right ventricle. The final calculations were performed with the remaining 112 tissue enhancement curves.

### Correction of the arterial input function

Before the determination of *MBF*, the correction procedure of the high concentration AIF was performed using the traditional dual bolus and the modified dual bolus methods. The modified dual bolus method is based on the Steward-Hamilton principle [23].

1 $$ D=Q\times AUC $$

where *D* = mass of the injected tracer, *Q* = cardiac output and *AUC* = area under the “first pass” concentration curve (i.e. AIF). According to this principle, the areas under low and high concentration AIF are proportional to their contrast agent concentrations, and cardiac output during the measurement of AIF. The cardiac output is product of stroke volume, and heart rate (*HR*). In this study, the stroke volume was assumed to stay constant, but heart rate variation in between the low and high concentration contrast agent injections was taken into account. The ratio of areas under low and high concentration AIFs can be expressed:

2 $$ {AUC}_{ratio}=\frac{D_{low}}{D_{high}}\frac{HR_{low}}{HR_{high}}. $$

The values of *AUC* for low and high concentration AIFs were determined by fitting the gamma variate function to the data points of the AIF, and by calculating the area under the fitted curve. Then, in the modified dual bolus method, the AIF of high concentration contrast agent injection was corrected mathematically (Please see Additional file 2). Effectively, the height of the AIF was raised until the ratio of low concentration and high concentration AIF areas were identical to *AUC*_*ratio*_. The full width at half maximum (FWHM) of the AIF peak was maintained constant.

For comparison, the AIF correction was conducted also by using the standard dual bolus method [13]. Every signal-intensity (SI)- data point was multiplied by 21, which was the concentration ratio of high and low concentration injections. Then the high concentration AIF was replaced with the scaled low concentration AIF which was placed to begin at the same time as the high concentration AIF.

In the modified dual bolus method, the actual high concentration AIF was corrected as described above. The main goal was to maintain the FWHM and shape of the primary AIF and correct only the height of the AIF. This method is not affected by potential variation in the HR between the different injections. In the dual bolus method, the actual high concentration AIF was removed, and it was replaced by an upscaled low concentration bolus AIF. This method does not take into account the variation of HR and is thus susceptible to differences in *HR* between the injections.

Modified dual bolus technique is described in detail in Additional file 2.

The corrected AIF-curves were used in further calculations of *MBF.*

### MRI MBF calculations

The relationship between AIF and the contrast agent concentration in tissue (output curve) can be described as the impulse response, *h(t)* using the convolution operation [24].

3 $$ {C}_t(t)={C}_b(t)\otimes h(t), $$

where *C*_*t*_*(t)* = contrast agent concentration in tissue, and *C*_*b*_*(t)* = contrast agent concentration in blood, i.e. AIF. MRI data is often noisy, causing deconvolution to produce an oscillating result. To avoid this, Tikhonov regularization technique [6, 25] was applied to stabilize the solution. The myocardial blood flow was determined from the calculated impulse response as *MBF* = *h*(*t* = 0); The whole procedure of regularized model independent deconvolution and determination of *MBF* from the calculated impulse response is fully described in Jerosch-Herold et al [6]. The advantage of the model independent deconvolution model is that it does not require the transfer function to follow any specific shape.

The values of perfusion reserve were calculated according to Eq. 4:

4 $$ Perfusion\kern0.17em reserve=\frac{MBF_{stress}}{MBF_{rest}}, $$

### Statistical analysis

IBM SPSS software version 22 (International Business Machines Corp. New York, NY, USA) was used in statistical analysis. Kolmogorov-Smirnov test was used to test whether the data followed the normal distribution. Because the data did not follow the normal distribution, the linear correlation coefficient between values of *MBF* between the modified dual bolus method and PET, as well as between the traditional dual bolus method and PET was determined using Spearman correlation analysis. Intraclass correlation analysis was applied when invesigating the conformity between values of *MBF* determined with the modified dual bolus method and PET, as well as between those determined using the traditional dual bolus method and PET. Furthermore, Bland-Altman analysis was performed to evaluate the agreement between the modified dual bolus method and PET, and between the traditional dual bolus method and PET. Because the data was not normally distributed, Wilcoxon signed rank test was used to test the statistical significance of difference in values of *MBF* in stress and rest, and the perfusion reserve determined with PET, the modified dual bolus method and the dual bolus methods.

## Results

The mean values of *MBF* in stress and rest determined with PET, modified dual bolus and dual bolus methods are presented in Table 2. The mean values of *MBF* determined with the modified dual bolus method were close to those determined with PET. Instead, there was bigger difference between the dual bolus method and PET in both stress and rest.

**Table 2:** The values of *MBF* (mean ± SD) in stress and rest, and the perfusion reserve determined with PET, the modified dual bolus method and the dual bolus method

	*MBF*_*stress*_ (ml/g/min)	*MBF*_*rest*_ (ml/g/min)	Perfusion reserve^b^
PET (mean ± SD)	4.44 ± 0.82	1.49 ± 0.36	3.13 ± 0.51
Modified dual bolus (mean ± SD)	3.63 ± 0.66^a^	1.36 ± 0.56^a^	2.83 ± 1.12
Dual bolus (mean ± SD)	2.17 ± 0.91^a^	2.01 ± 2.36	2.13 ± 1.25

^a^Statistically significant difference compared with PET (*p* ≤ 0.01)

Wilcoxon signed rank sum test

^b^ The values of perfusion reserve were determined only for those segments where both stress and rest values of *MBF* were available

**Fig. 2:**
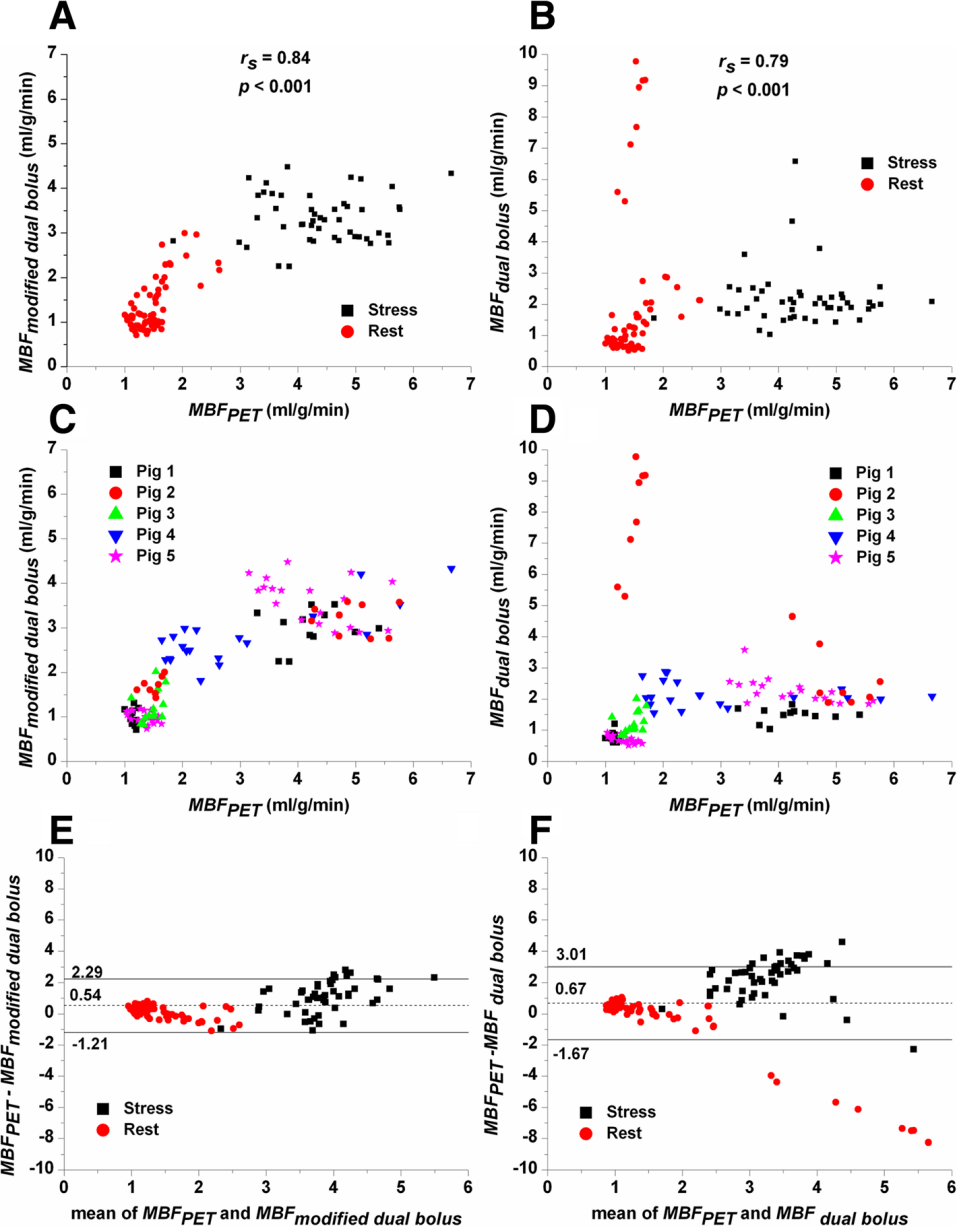
The modified dual bolus method and the dual bolus method compared with PET. The values of *MBF* during stress (square) and at rest (circle) determined with PET compared with *MBF* determined with (**a**) modified dual bolus method and (**b**) dual bolus method. The same data is presented in (**c**) and (**d**) respectively, but reporting the values of *MBF* separately for each animal. In the rest study of pig#2, the change of heart rate between the injections of the low and high concentrations of contrast agent and this introduced variation in the input functions, and subsequently in the calculated *MBF* values. Bland-Altman plots of differences between (**e**) *MBF*_*PET*_ and *MBF*_*modified dual bolus*_ and (**f**) *MBF*_*PET*_ and *MBF*_*dual bolus*_ vs. the mean of measurements with the corresponding methods. The limits of agreement (*mean* ± 1.96*SD*) are also presented. Wilcoxon signed rank test was used to study the statistical significance of difference between *MBF*_*PET*_ and *MBF*_*modified dual bolus*_ and (*MBF*) *MBF*_*PET*_ and *MBF*_*dual bolus*_. Statistically significant difference (*p* < 0.001) was found in both cases

The values of *MBF* determined with the modified dual bolus method were correlated with the values of *MBF* estimated with PET (*r*_*s*_ = 0.84, *p* < 0.0001) (Fig. 2a and c). The correlation between the values of *MBF* determined with the traditional dual bolus method and those with PET was slightly weaker (*r*_*s*_ = 0.79, *p* < 0.0001) (Fig. 2b). A very good intraclass correlation (ICC = 0.85, *p* < 0.0001) was found between the *MBF* values determined with the modified dual bolus method and PET, whereas the intraclass correlation between the traditional dual bolus method and PET was poor (ICC = 0.07, *p* = 0.227). The values of *MBF* at rest determined with the dual bolus and modified dual bolus methods were relatively similar, although statistically significantly different, compared to those determined with PET (Fig. 2a and c). The values of *MBF* during stress were slightly underestimated with the modified dual bolus method (Figs. 2a and c), but with the dual bolus method there was an even greater underestimation of *MBF* (Fig. 2b and d). In pig #2, *MBF* determined with the traditional dual bolus method differed markedly from the corresponding value determined with PET and the modified dual bolus method (Fig. 2d). A major difference in the heart rates (51 bpm vs. 75 bpm, Additional file 1: Table S1.1) between the low and high concentration AIFs occurred in this pig; this caused for the difference in the *MBF values determined* using the traditional dual bolus method. Bland-Altman plots (Fig. 2e and f) demonstrated better agreement between the modified dual bolus method and PET than between the dual bolus method and PET.

## Discussion

Quantification of the *MBF* offers the possibility to diagnose heart diseases and to follow-up the effectiveness of treatments. One advantage of MR imaging is the possibility to study both anatomy and function of the heart during the same session. The main challenge in the quantification of the *MBF* with contrast agent enhanced MRI has been the T1-effect, which causes error in the AIF [26] leading to inaccuracy in the determination of *MBF*. For this reason, it is important to devise some way to control the T1-effect during a contrast agent enhanced MRI study.

In the present study, the conventional dual bolus method [13] and the modified dual bolus method described previously [14] were compared with PET in the determination of myocardial perfusion in a porcine model of cardiac ischemia. The PET and contrast agent enhanced MRI studies were performed on five pigs during the same session, with the same scanner. This was a great advantage ensuring that the same local perfusion was measured with both methods; it also made it possible to utilize PET *MBF* values as a reference against which to compare the dual bolus MRI methods.

A strong correlation was found between the *MBF* determined with the modified dual bolus MRI method and PET. *MBF* determined with the modified dual bolus and the traditional dual bolus methods were very similar at rest, except in pig#2. In that specific case, the heart rate at rest was lower during the low concentration pre-bolus injection than during the high concentration injection (51 bpm vs. 75 bpm). The change in the heart rate is considered in the modified dual bolus method (Eq. 4 in Additional file 2), but ignored in the traditional dual bolus method. This means that the size and shape of the AIF in the modified dual bolus method and in the traditional dual bolus method are clearly different, as shown previously [14], leading to differences in the calculated *MBF* values. To find out the difference between the traditional and the modified dual bolus method in case of minor variation in heart rate, the average values of *MBF* were recalculated. In these recalculations one rest study of the pig with large variation in heart rate was excluded. These values are present in Additional file 3: Table S3.1. As expected, the performance of the dual bolus method improved after the exclusion. However, the modified dual bolus method still outperformed the dual bolus method.

The modified dual bolus method produces more reliable *MBF* values, as highlighted in the better intraclass correlation with the *MBF* values determined using PET and by the better agreement between PET and the modified dual bolus method than between PET and the traditional dual bolus method. Furthermore, the mean values of *MBF* determined with the modified dual bolus method are in better agreement with PET than those determined with the dual bolus method. However, with both MRI methods *MBF*_stress_ values, as well *MRI*_*rest*_ values determined with modified dual bolus method were statistically significantly different from those determined with PET. Only the mean value of *MBF*_*I*_ determined with the dual bolus method was not significantly different from that determined with PET. This can be explained by great variation in *MBF*_*rest*_ values determined with the dual bolus method.

Significant variation in heart rate was present only in one of the pigs at rest, i.e. 1/9 of all data (Additional file 1: Table S1.1). In this study, the pigs were anesthetized and connected to a respirator, stabilizing the heart rate. It would have been possible to reduce the heart rate variation by administering medication (for example, β-blockers). However, in an earlier study [14] a difference in *K*^*trans*^ was found in cases where the heart rate or duration of AIF was different between the low and high concentration injections. For this reason, we wanted to determine which method would also deliver reliable results when there were variations in the heart rate. In the clinical setting, patients commonly have irregular heart rates. Thus, it is important to have a method that is insensitive to heart rate variation when determining myocardial perfusion.

Another advantage of the modified dual bolus method is that the correction of the AIF is performed on the high concentration injection data. Furthermore, only the non-linear part of the data, i.e. the peak of the AIF is corrected. Thus, the data preceding and following the peak of the AIF are left unchanged. This correction method preserves the dynamics of the high concentration bolus, which generates the tissue enhancement curves that are analyzed. In comparison, the traditional dual bolus method uses only the low concentration pre-bolus data in the determination of the AIF, and any random variations between the two cases, including those not related to myocardial perfusion may introduce errors into the calculated values.

The values of *MBF* in stress determined with the modified dual bolus technique were slightly lower than those determined with PET. However, the modified dual bolus method was found to be more reliable than the dual bolus method in high values of *MBF*. The values of *MBF* determined with the traditional dual bolus method were lower than those estimated with PET. This is also reflected in the higher intraclass correlation between the modified dual bolus method and PET than between the traditional dual bolus method and PET. Furthermore, Bland-Altman analysis revealed good agreement between the *MBF* values determined with PET and the modified dual bolus method. Instead, deviations were noted with the traditional dual bolus method, especially at higher *MBF* values. The values of *MBF* in stress were lower when determined with both MR methods compared with those determined with PET. This is possibly due to the pharmacokinetic nature of Gd -contrast agent. While extraction factor of ^15^O is not limited by *MBF*, the extraction fraction of Gd- contrast agent is dependent on *MBF*, being lower in stress [27]. This causes the underestimation of *MBF* in stress in MR compared with PET. Another explanation to this difference is the cyclic variation of *MBF* in stress. Motwani et al. [28] reported 25% higher *MBF* during end-diastole than in end-systole. Depending on the heart rate, the moment of image data acquisition in MR may be during the end-systole or early-diastole. PET frames span several seconds, averaging over the entire cardiac cycle. Therefore, depending on how much of the MR imaging data is acquired during the end-systole, there may be additional underestimation of *MBF* in stress.

In addition to inconsistencies in the dynamics between the low and high concentration injections, this deviation may be due to the high concentration ratio (1/21) between the high and low concentration pre-bolus injections. The traditional dual bolus method has been shown to be functional with the dilution/contrast agent ratio of 1/10 [29, 30]. In an earlier study [14] a dilution concentration of 1/8 was used. This was reported to work well, but to cause negligible (yet detectable) enhancement into myocardium, which we wanted to avoid in this study. For that reason, we examined different contrast agent dilutions, and found the dilution ratio of 1/21 optimal for the low concentration pre-bolus.

In clinical practice, the AHA segment model based on three short axis slices: basal, mid and apical is commonly used for segmental analysis. However, pigs often have much higher heart rate compared with human patients. Therefore, it would have been impossible to acquire three image slices during one cardiac cycle. 8 mm slice thickness was considered to yield sufficiently high signal to noise ratio. On the other hand, the slice was thin enough to avoid artefacts due to the partial volume effect in the apical part of the left ventricle. In the AHA segment model, basal and middle slices are divided into six segments, and apical slice into four segments. However, to avoid the averaging of *MBF* inside the large segments, the smaller segments were used to enable measurement of the spatial *MBF* as accurately as possible.

Propofol and Fentanyl were used to maintain anaesthesia. Propofol has a tendency to decrease the systemic vascular resistance and arterial blood pressure, and therefore cause increase of cardiac output. Fentanyl prevents the decrease of arterial blood pressure. Combination of Propofol and Fentanyl together does not affect haemodynamic variables and is therefore suitable to be used with animals involved in cardiovascular research [31]. The anaesthesia was maintained static during PET and MR imaging.

The present experimental setup has some shortcomings. The goal of this study was to compare the modified dual bolus method against PET which is the gold standard in the determination of *MBF*. The most reliable way to carry out this comparison is to perform the PET and MRI studies using a PET-MRI scanner. The only PET-MRI scanner that was available for us, was equipped with 3 T MRI. The dark rim artefact was very common and numerous regions could not be quantified [22]. One possible reason of dark rim artefact may be the resolution of MR images. It is known that this Gibbs ringing artefact arises from the truncation of the higher spatial frequencies of the true object. In this study the in-plane pixel size was 2.7 mm × 3.8 mm. If the spatial resolution could be enhanced to < 2.5 mm, this artefact could be minimized. It is also possible, that the dark rim artefact is associated with the high concentration of the contrast agent bolus and motion in the left ventricle cavity. The high concentration of the contrast agent decreases the T1 relaxation time, but also the T2* relaxation time. The decrease of the T2* relaxation time is related to the susceptibility effect of the high concentration Gd-contrast. If it were possible to shorten the echo time, this artefact would likely be minimized. Another solution to minimize the dark rim artefact might be the use of a lower dose of the contrast agent, although one cannot lower the dose too much as this would lead to a loss of detection of the enhancement in low perfusion areas of interest. The susceptibility artefact is more pronounced at 3 T than at 1.5 T, and could therefore be reduced by using a 1.5 T scanner instead of a 3 T scanner. Thus, either using a 1.5 T scanner or a possibly a lower contrast agent concentration at 3 T is recommended as rejection rate of 50% of myocardial segments is not acceptable in clinical practice.

The bottleneck stent was inserted to the left anterior descending coronary artery providing most of the blood supply to the interventricular septum. Therefore, septum was the most probable area for the ischemia. As a results the local ischemia can be seen in some segments as lower value of *MBF* in stress. However, there is individual variation in *MBF* between the pigs anyway.

The contrast agent injections were performed manually, because at the time of the experiments, a 3 T compatible power injector was not available in our laboratory. The duration of the injection (contrast agent + 15 ml of saline) was approximately 5–7 s, which translates to a rate of about 3.5 ml/s. A higher flow rate would have been desirable. Too low flow rate (< 3 ml/s) has been shown to cause slower upslope of signal intensity in myocardium. This may lead to underestimation of myocardial perfusion. However, the flow rate > 3 ml/s has been found to ensure reliable results [32]. While the manual injection introduces variation, we found the modified dual bolus method to work well also in this suboptimal situation.

Since there is no method to measure the stroke volume during the dynamic imaging series, we assumed that it would remain constant even when heart rate changed. However, the relation between the heart rate and stroke volume is very complicated [33, 34]. At rest, the stroke volume has been found to decrease when the heart rate increases [35, 36]. However, decrease of the stroke volume does not completely compensate for the effect of increased heart rate, which results in a slight increase in the cardiac output. On the other hand, during stress, the stroke volume increases when heart rate increases [37]. This increases the cardiac output more than would be indicated by the change in heart rate per se. Thus, the correction of cardiac output based only on heart rate overestimates the change at rest and underestimates the change at stress, but is still reasonable and, in both cases, better than no correction at all. According to the present results, one can significantly improve the reliability of determination of *MBF* by taking into account the variation in the heart rate.

In this study, local myocardial ischemia was induced in the pig heart using a constricted bare metal stent. The purpose of this stent was to evoke a local ischemia and therefore lead to a wide range of *MBF* values. However, it is possible that in an extreme case, a local infarct could have occurred.

## Conclusions

In conclusion, a strong correlation was found between the modified dual bolus method and PET. The modified dual bolus method was found to be more reliable than the conventional dual bolus method, especially if there were variations in the heart rate, or if there were any inconsistencies between the low and high concentration injections. However, in terms of quantification, it should be noted that the difference between the *MBF* values estimated with either of the two MRI-based dual-bolus methods and those estimated with the gold-standard PET method were statistically significant.

## Additional files

Additional file 1: The segments included in analysis and heart rates during perfusion imaging. (PDF 7 kb)

Additional file 2: Modified dual bolus method. (PDF 44 kb)

Additional file 3: Recalculated mean values of *MBF*, when data of pig #2 rest study is excluded. (PDF 6 kb)

## Data Availability

The datasets generated and analysed during the current study are not publicly available since they are part of larger material that is still under further analysis for future publications. However, the data is available from the corresponding author on reasonable request.

## Abbreviations

AIF: Arterial input function
*CO*: Cardiac output
*HR*: Heart rate
*K*^*trans*^: Unidirectional transfer constant
LV: Left ventricle
*MBF*: Myocardial blood flow
PET: Positron emission tomography
ROI: Region of interest
SI: Signal intensity
T1-TFE: 2D saturation recovery segmented gradient recalled echo T1-weighted
TAC: Time-activity curve
TE: Time to echo
TR: Time to repetition
VOI: Volume of interest
