# Supplementary material for: Quantification of porcine myocardial perfusion with modified dual bolus MRI – a prospective study with a PET reference
Source: BMC Med Imaging. 2019 Jul 26;19:58. doi: 10.1186/s12880-019-0359-8 (PMC6660956; doi:10.1186/s12880-019-0359-8)
Supplement: Supplementary file 1 — The segments included in analysis and heart rates during perfusion imaging. (PDF 7 kb) [file 12880_2019_359_MOESM1_ESM.pdf]

Additional file 1

The segments included in analysis and heart rates during perfusion imaging

Table S1.1. The segments included in results and heart rates (*HR*) during high and low concentration contrast agent injections in stress and rest for each pig.

Pig	Included segments*, stress	Included segments*, rest	*HR* stress low/high	*HR* rest low/high
#1	Basal: 1, 5-11<br>Apex: 1,7-8	Basal 1,8-9, 15<br>Apex 1, 3-8	112/110	100/100
#2	Basal: 6-9, 13-14<br>Apex: 4, 7-8	Basal 6-8, 14-15<br>Apex 4, 7-8	76/72	51/75
#3	-	Basal 1,8,10-16<br>Apex 4-8	-	76/75
#4	Basal: 7, 13-15<br>Apex: 2-4, 6-7	Basal: 1-3, 7-8, 13-16<br>Apex: 4, 7-8	62/59	63/63
#5	Basal: 4-9, 11, 13-16<br>Apex: 1-8	Basal: 1-6, 10-16<br>Apex: 1-3, 5-8	64/57	65/64

*Numbers of the segments are present in main manuscript in figure 1.
