# Supplementary material for: Quantification of porcine myocardial perfusion with modified dual bolus MRI – a prospective study with a PET reference
Source: BMC Med Imaging. 2019 Jul 26;19:58. doi: 10.1186/s12880-019-0359-8 (PMC6660956; doi:10.1186/s12880-019-0359-8)
Supplement: Supplementary file 2 — Modified dual bolus method. (PDF 44 kb) [file 12880_2019_359_MOESM2_ESM.pdf]

Additional file 2

The modified dual bolus method

Before the determination of *MBF*, the correction procedure of the high concentration AIF was performed. The first step in the correction procedure was baseline correction, which was performed for all low concentration pre-bolus AIFs, for high concentration AIFs, and for tissue enhancement curves. We applied the Steward-Hamilton principle,

$$D = CO \times AUC\,, \tag{1}$$

where D = mass of the injected tracer, CO = cardiac output and AUC = area under the first pass concentration curve (i.e. AIF) [A1]. The peak areas of low concentration pre-bolus and high concentration AIFs were determined by calculating the area under the gamma variate function fitting [A2, A3]

$$C(t) = K(t - AT)^{\tilde{}}\, e^{-(t-AT)/}\quad , \tag{2}$$

where t = time after contrast agent injection, K = scaling factor, AT = time delay between contrast agent injection and occurrence in the region of interest (ROI), while α and β are fitting parameters. Because the area under the dye curve is proportional to dye concentration [A4], the ratio of areas under the dye curves (AUC_{ratio}) of low concentration pre-bolus imaging series and high concentration image series can be written as follows

$$AUC_{ratio} = \frac{D_{low}}{D_{high}} \frac{CO_{low}}{CO_{high}}, \tag{3}$$

where D_{low} is contrast agent dose (ml) in the prebolus injection and D_{high} in high concentration contrast agent injections. CO_{low} and CO_{high} are cardiac outputs during the prebolus and with the

injections of high concentrations of contrast agent, respectively. Cardiac output is the product of stroke volume and heart rate (*HR*). It is assumed here that the stroke volume remains constant and thus the determination of cardiac output simplifies into the determination of heart rate. Therefore, the equation (4) can be written as follows

$$AUC_{ratio} = \frac{D_{low}}{D_{high}} \frac{HR_{low}}{HR_{high}}. \tag{4}$$

If no non-linearity occurs at high concentration AIF, the ratio of areas under the high concentration AIF and low concentration pre-bolus AIF is equal to AUC_{ratio}. As expected, saturation in all high concentration AIF curves was seen. Because of this, a correction procedure for the high concentration AIF curves was performed: the AIF curve was manipulated by means of constrained gamma variate fitting. The high concentration AIF was fitted by imposing additional constraints: 1) the area of the fitted curve had to equal the area of the pre-bolus fit curve * AUC_{ratio}, i.e. to fulfil the condition presented in equation (4) and 2) the peak width (FWHM) had to match the FWHM of peak of the unmodified high concentration AIF. In other words, AUC_{ratio} was used in the cost function of the direct numerical fit (fminsearch function in Matlab) of the AIF to ensure the correct area ratio of the low and high concentration curves. For this, the AIFs of the low and high concentration contrast agent injections were first directly fitted with the equation (2) to determine the areas of the *measured* low and high concentration AIFs (AUC_{low} and AUC_{high}). Subsequently the unadjusted fits and the AUC_{ratio} were used in the cost function of a second fit of the high concentration AIF by requiring the above conditions to be met by

$$argmin\left\{ \sim \left\| \int \hat{C}(t) - (1+©)AUC_{ratio}AUC_{low} \right\| + \quad \left\| \hat{C}(T) - C(T) \right\| \right\}, \qquad (5)$$

where $\hat{C}(t)$ is the adjusted high concentration AIF peak fit, $C(t)$ is the unadjusted high concentration fit from previous step, T is the last fitted point of the peak (see Fig. A1) and α, β and κ are empirical weighting factors, which balance the conditions since they effectively impose constraints along the same dimension of signal intensity. In practice, adjusted fits with correct final AUC_{ratio} were obtained by setting α, β and κ to approximately α, κ=0.2 and β=1-α. Furthermore, every high concentration AIF fit was manually confirmed.

By imposing these two factors in the fitting, the method did not affect the location or FWHM of the AIF. The initial data points before and after the AIF peak were kept unchanged. The correction procedure is visualised in figure A1.

Details of a similar procedure for manipulation of high concentration AIF are presented in Husso *et al* [A5].

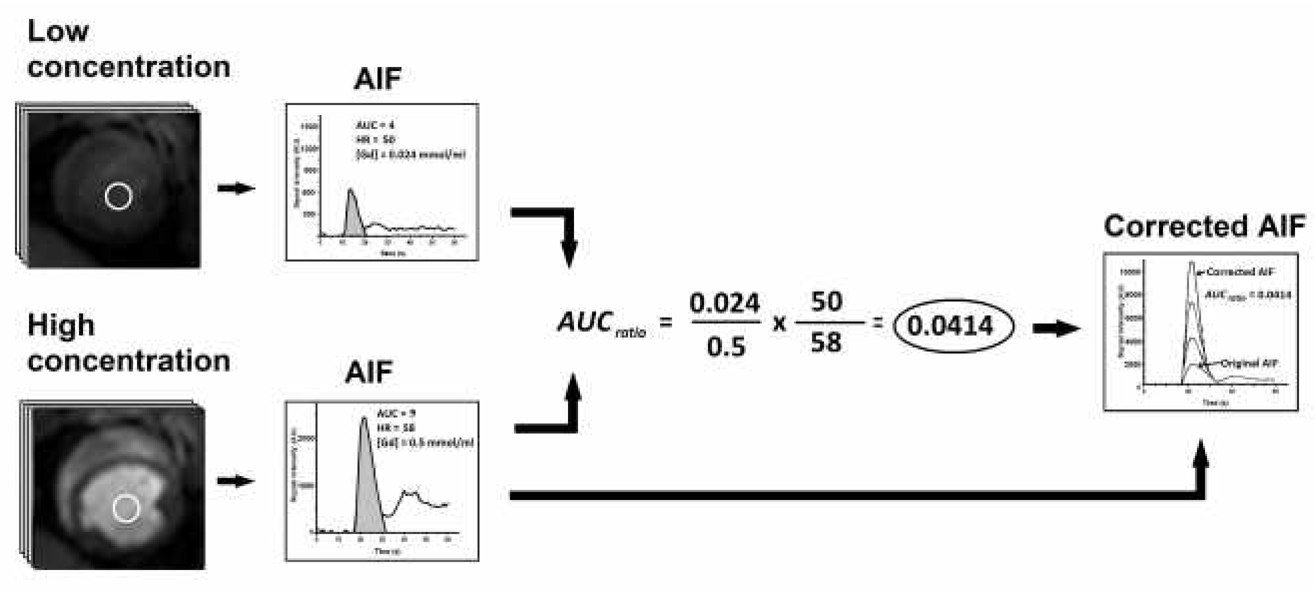

Figure S2.1 First, the arterial input functions (AIF) for low and high concentration series were determined: The signal intensity vs. time curves from left ventricle cavity (circle) were obtained. After that, the areas under input curve (AUC) were calculated for both AIF curves. The ratio of areas under AIF curves (AUC_{ratio}, equation 4) was calculated using heart rates and concentrations of contrast agent injections. Then, the original high concentration AIF was raised until the ratio under low and high concentration AIFs was equal to AUC_{ratio}.

References

A1. Zierler KL. Equations for Measuring Blood Flow by External Monitoring of Radioisotopes. Circ Res 1965;16:309-21.

A2. Thompson HK, Starmer CF, Whalen RE, McIntosh HD. Indicator Transit Time Considered as a Gamma Variate. Circulation 1964;14:502-15.

A3. Starmer CF, Clark DO. Computer computations of cardiac output using the gamma function. J Appl Physiol 1970;28:219-20.

A4. Bassingthwaighte JB, Ackerman FH. Mathematical linearity of circulatory transport. J Appl Physiol 1967;22:879-88.

A5. Husso M, Sipola P, Kuittinen T, Manninen H, Vainio P, Hartikainen J, *et al.* Assessment of myocardial perfusion with MRI using a modified dual bolus method. Physiol Meas 2014;35:533-47.
