# Supplementary material for: Quantification of porcine myocardial perfusion with modified dual bolus MRI – a prospective study with a PET reference
Source: BMC Med Imaging. 2019 Jul 26;19:58. doi: 10.1186/s12880-019-0359-8 (PMC6660956; doi:10.1186/s12880-019-0359-8)
Supplement: Supplementary file 3 — Recalculated mean values of MBF, when data of pig #2 rest study is excluded. (PDF 6 kb) [file 12880_2019_359_MOESM3_ESM.pdf]

Additional file 3

Recalculated mean values of *MBF*, when data of pig #2 rest study is excluded.

Table S3.1. The mean values of MBF (mean±SD) in stress and rest determined with PET, the modified dual bolus method and the dual bolus method. Data of pig #2 rest study is excluded.

	MBF_{stress} (ml/g/min)	MBF_{rest} (ml/g/min)
PET (mean ± SD)	4.44 ± 0.82	1.49 ± 0.38
Modified dual bolus (mean ± SD)	3.63 ± 0.66*	1.36 ± 0.63
Dual bolus (mean ± SD)	2.17 ± 0.91*	1.19 ± 0.66*

*Statistically significantly different compared to PET ($p \leq 0.01$), Wilcoxon signed rank sum test.
